# Complete mitochondrial genome of *Gloydius saxatilis* (Viperidae: Crotalinae) from Korea

**DOI:** 10.1080/23802359.2021.1878957

**Published:** 2021-02-17

**Authors:** Yun Sun Lee, Min Seock Do, Hye Sook Jeon, Sang-Cheol Lee, Ji-Hwa Jung, Jae-Hwa Suh, Junghwa An

**Affiliations:** a Conservation Genome Resource Bank for Korean Wildlife, College of Veterinary Medicine, Seoul National University, Seoul, Republic of Korea; b Animal Resources Division, National Institute of Biological Resources, Incheon, Republic of Korea; c Department of Biology, Incheon National University, Incheon, Republic of Korea; d Department of Forest Sciences, Seoul National University, Seoul, Republic of Korea

**Keywords:** *Gloydius saxatilis*, Viperidae, viper, mitochondrial genome, next-generation sequencing

## Abstract

In this study, we sequenced the complete mitochondrial genome of *Gloydius saxatilis* using Illumina next-generation sequencing. The total length of the mitogenome was 17,223 bp, and contained 13 protein-coding genes (PCGs), two ribosomal RNAs (rRNAs), 22 transfer RNAs (tRNAs), two non-coding control regions (CRs), and the origin of light (OL)-strand replication. The genome structure and order of the genes were similar to other Crotalinae species. Phylogenetic analysis based on the 13 concatenated PCGs indicated that *G. saxatilis* closely related to *G. intermedius* and, *G. shedaoensis.*

*Gloydius saxatilis* (Emelianov, 1937) is a venomous pit viper species endemic to Asia (Russia, China, and the Korean Peninsula) and belongs to the family Viperidae. Despite being assessed as Least Concern in IUCN Red List because of its large distribution in forest areas and mountainous zones, *G. saxatilis* in Korea has been decreasing due to poaching. The genetic information and biological data about this species are lacking. Also, many researchers in Korea used *G. intermedius* as an alternative name of *G. saxatilis* (David and Vogel 2015). In this study, we determined the complete mitogenome of *G. saxatilis*.

*Gloydius saxatilis* tissue samples (NIBR0000625204) were collected from Samcheok-si, Gangwon-do, South Korea (37.109 N, 129.180 E) and stored in the National Institute of Biological Resources (NIBR: https://www.nibr.go.kr) in Incheon, South Korea. Genomic DNA was extracted using the DNeasy Blood and Tissue Kit (Qiagen, Germantown, MD). Average library insert size of 550 bp was construction and next-generation-sequencing was performed on the IlluminaNovaSeq6000 platform at DNA Link Inc. (Seoul, South Korea). The raw data were generated with 150 bp paired-end lengths. De-novo assembly was performed using GetOrganelle version 1.6.4 (Jin et al. 2020). Gene structures of the mitochondrial genome were annotated with GeSeq (Tillich et al. 2017), while tRNA detection was predicted by ARAGORN version 1.2.38 (Laslett and Canback 2004) and tRNAscan-SE version 2.0.5 (Lowe and Chan 2016). Mitogenome sequence of *G. saxatilis* was submitted in GenBank (accession no. MW143075).

The complete *G. saxatilis* mitogenome sequence was 17,223 bp in length and contained two non-coding control regions (CRs), 13 protein-coding genes (PCGs), two f ribosomal *RNA* genes (rRNAs), 22 transfer *RNA* genes (tRNAs), and origin of light (OL)-strand replication, similar to other Crotalinae species (Supplementary Table S1). The A-T content (58.2%) was significantly higher than the G-C content (41.8%) following base-pair composition: A – 32.37%, C – 28.76%, G – 13.00%, and T – 25.87%. Most of the genes in *G. saxatilis* were located on the heavy strand (H-strand), except for the one PCG (*ND6*) and eight tRNAs (*tRNA-Pro*, *Gln*, *Ala*, *Asn*, *Cys*, *Tyr*, *Ser*, and *Glu*), located on the light strand (L-strand). Ten PCGs started with ATG; the exceptions are *COX1*(GTG), *ND1*(ATA), and *ND3* (ATT). On the contrary, only six PCGs terminated with the complete vertebrate mitochondrial stop codons TAA (*ATP8*, *ATP6*, *ND4L*, and *ND5*), and AGA (*COX1* and *ND4*). The other seven genes had incomplete stop codons, ending as CAT (*ND2*, *ND6*, and *COX3*), AAT (*ND3* and *ND1*), AGT (*COX2*), and CCT (*CYTB*).

The maximum-likelihood (ML) tree with 1000 replicates was constructed using MEGA X (Kumar et al. 2018). The general-time-reversible model (GTR) with Gamma distributed (+G) was selected as best-fit-model. The phylogenetic tree was based on the 13 concatenated PCGs and mitogenome data corresponding to 35 individuals of 27 Crotalinae species obtained from GenBank. This phylogenetic analysis indicated that *G. saxatilis* closely related to *G. intermedius* and, *G. shedaoensis* (Figure 1). In conclusion, this study provides a new additional high-quality mitogenome database for further evolutionary and molecular research on Crotalinae.

**Figure 1.**
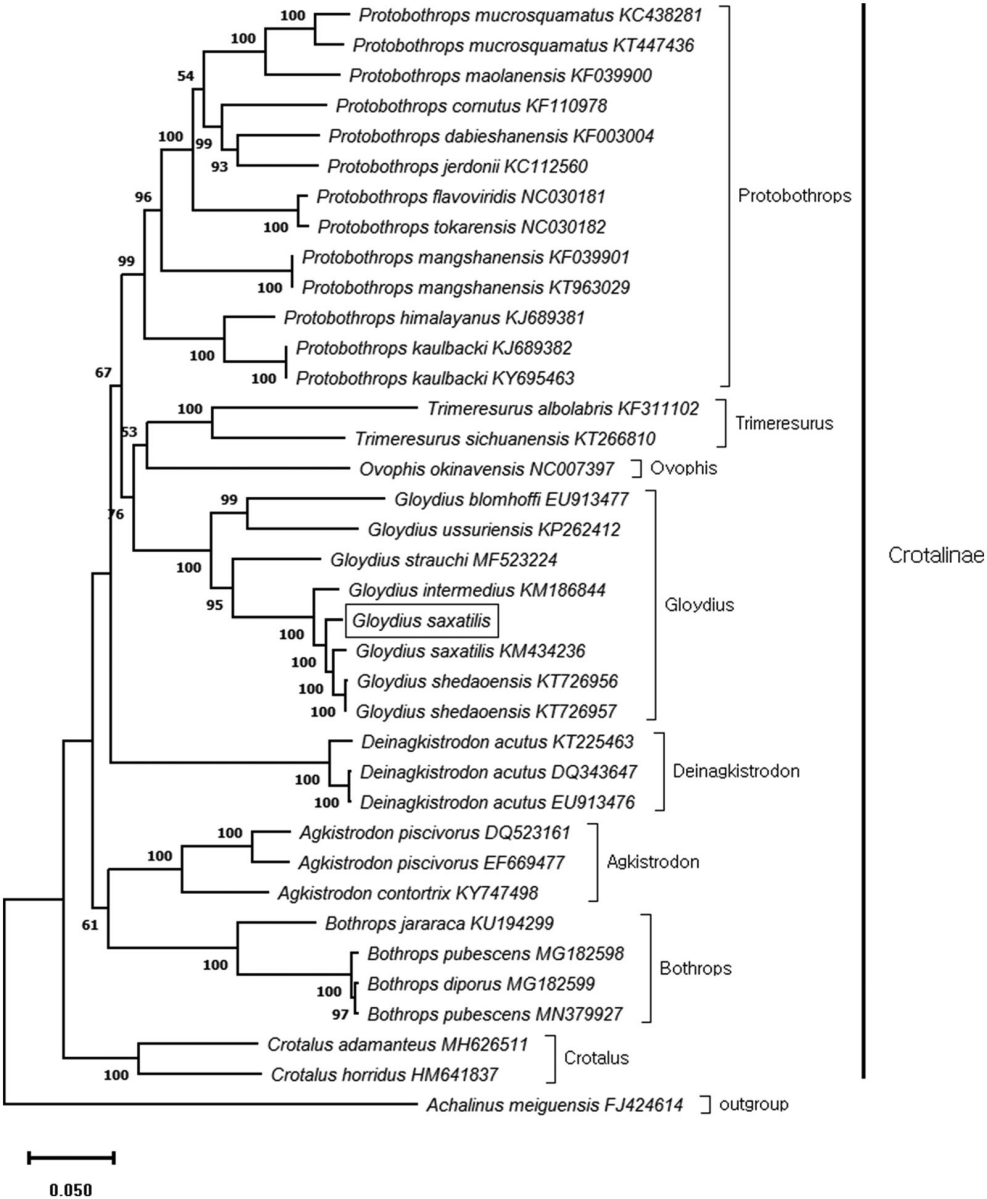
Maximum likelihood (ML) phylogenetic tree based on 13 concatenated protein-coding genes of 36 viper mitogenomes. The Sichuan odd-scaled snake (*Achalinus meiguensis*) was used as an out group. Node labels indicate bootstrap values. The mitogenome of *Gloydius saxatilis* (square box) was determined in this study.

## Supplementary Material

Supplemental Material

## Data Availability

The genome sequence data that support the findings of this study are openly available in GenBank of NCBI at (https://www.ncbi.nlm.nih.gov) under the accession no. MW143075.
