# Supplementary material for: Complete mitochondrial genome of Gloydius saxatilis (Viperidae: Crotalinae) from Korea
Source: Mitochondrial DNA B Resour. 2021 Feb 17;6(2):645–7. doi: 10.1080/23802359.2021.1878957 (PMC7894462; doi:10.1080/23802359.2021.1878957)
Supplement: Supplemental Material [file TMDN_A_1878957_SM1346.docx]

Supplementary Table S1. List of *Gloydius saxatilis* mitogenome annotation.

| Name | Type | Minimum | Maximum | Length | Direction |
| --- | --- | --- | --- | --- | --- |
| tRNA-Phe | tRNA | 1 | 57 | 57 | forward |
| 12S rRNA | rRNA | 58 | 968 | 911 | forward |
| tRNA-Val | tRNA | 969 | 1031 | 63 | forward |
| 16S rRNA | rRNA | 1032 | 2509 | 1478 | forward |
| ND1 CDS | CDS | 2510 | 3470 | 961 | forward |
| tRNA-Ile | tRNA | 3471 | 3536 | 66 | forward |
| tRNA-Pro | tRNA | 3539 | 3602 | 64 | reverse |
| control region D-loop | D-loop | 3603 | 4629 | 1027 | forward |
| tRNA-Leu | tRNA | 4630 | 4702 | 73 | forward |
| tRNA-Gln | tRNA | 4703 | 4771 | 69 | reverse |
| tRNA-Met | tRNA | 4772 | 4834 | 63 | forward |
| ND2 CDS | CDS | 4835 | 5862 | 1028 | forward |
| tRNA-Trp | tRNA | 5865 | 5930 | 66 | forward |
| tRNA-Ala | tRNA | 5931 | 5995 | 65 | reverse |
| tRNA-Asn | tRNA | 5996 | 6068 | 73 | reverse |
| origin of light-strand (OL) | rep_origin | 6069 | 6107 | 39 | forward |
| tRNA-Cys | tRNA | 6108 | 6167 | 60 | reverse |
| tRNA-Tyr | tRNA | 6168 | 6228 | 61 | reverse |
| COX1 CDS | CDS | 6230 | 7831 | 1602 | forward |
| tRNA-Ser | tRNA | 7822 | 7889 | 68 | reverse |
| tRNA-Asp | tRNA | 7890 | 7952 | 63 | forward |
| COX2 CDS | CDS | 7953 | 8637 | 685 | forward |
| tRNA-Lys | tRNA | 8638 | 8699 | 62 | forward |
| ATP8 CDS | CDS | 8700 | 8864 | 165 | forward |
| ATP6 CDS | CDS | 8855 | 9535 | 681 | forward |
| COX3 CDS | CDS | 9535 | 10318 | 784 | forward |
| tRNA-Gly | tRNA | 10319 | 10379 | 61 | forward |
| ND3 CDS | CDS | 10380 | 10722 | 343 | forward |
| tRNA-Arg | tRNA | 10723 | 10786 | 64 | forward |
| ND4L CDS | CDS | 10787 | 11077 | 291 | forward |
| ND4 CDS | CDS | 11077 | 12414 | 1338 | forward |
| tRNA-His | tRNA | 12416 | 12477 | 62 | forward |
| tRNA-Ser | tRNA | 12478 | 12532 | 55 | forward |
| tRNA-Leu | tRNA | 12534 | 12605 | 72 | forward |
| ND5 CDS | CDS | 12607 | 14394 | 1788 | forward |
| ND6 CDS | CDS | 14390 | 14911 | 522 | reverse |
| tRNA-Glu | tRNA | 14912 | 14974 | 63 | reverse |
| CYTB CDS | CDS | 14975 | 16088 | 1114 | forward |
| tRNA-Thr | tRNA | 16089 | 16153 | 65 | forward |
| control region D-loop | D-loop | 16154 | 17223 | 1070 | forward |
